# RNA modification writers pattern in relation to tumor microenvironment and prognosis in prostate cancer

**DOI:** 10.3389/fgene.2022.1065424

**Published:** 2023-01-18

**Authors:** Xu Cheng, Xuanzi Yi

**Affiliations:** ^1^ Department of Urology, Xiangya Hospital, Central South University, Changsha, China; ^2^ Department of General Practice, The Third-Xiangya Hospital, Central South University, Changsha, China

**Keywords:** prostate cancer, RNA modification writers, immunotherapy, RM_score, prognosis

## Abstract

**Background:** RNA modifications are important in the study of epigenetic regulatory mechanisms in immune responses and tumorigenesis. When RNA writers are mutated or disrupted in expression, the genes associated with the pathways they modify are also disrupted and can activate or repress related pathways, affecting tumorigenesis and progression. However, the potential role of RNA writers in prostate cancer is unclear.

**Methods:** Based on data from three datasets, we describe 26 RNA writers that mediate gene expression and genetic mutation in prostate cancer and assess their expression patterns in 948 prostate cancer samples. Using principal component analysis algorithms, the RM Score was developed to quantify the RNA modification patterns of specific tumors.

**Results:** Two different categories were determined by unsupervised clustering methods, and survival analysis showed significant differences in OS prognosis between these two categories. Differentially expressed genes between the different categories were detected and the RNA writers-mediated scoring model RM_Score were constructed based on this. Also, the RM_Score was analyzed in relation to clinical characteristics, immune infiltration level, drug response, and efficacy of chemotherapy and immunotherapy. Those results confirm that multilayer alterations in epitope-modified RNA writers are associated with patient prognosis and with immune cell infiltration characteristics. Finally, we examined differentially expressed mRNA, lncRNA and miRNA between high and low RM_Score groups, based on which a ceRNA regulatory network was constructed.

**Conclusion:** This work is a comprehensive analysis of modified writers in prostate cancer and identified them to have a role in chemotherapy and immunotherapy.

## Introduction

In the male population worldwide, prostate cancer is the most common malignancy, and there is no effective treatment for advanced prostate cancer, particularly metastatic prostate cancer and castration-resistant prostate cancer (CRPC) (Siegel et al., 2021).% (Small and de Bono, 2011; Emmanuel et al., 2014; Royce et al., 2017).

In genetics, epigenetics is the study of stable and heritable phenotypes caused by changes in chromosomal sequence that do not alter gene sequences. In recent years, an increasing number of studies have demonstrated that RNA modification is a critical mechanism of epigenetic regulation and is involved in both physiological processes and disease development (Mo et al., 2014; Li et al., 2020; Ma et al., 2020; Yuan et al., 2020).

To fully understand the importance of post-transcriptional modifications, there is a need to explore the crosstalk between different patterns of these alterations. A few studies on *p*Ca have emphasized the importance of RNA alteration in carcinogenesis (Cai et al., 2019; Chen et al., 2021a). The majority of studies have concentrated on a limited number of genes, whereas RNA modification “writers” may form an important and complex network of cellular regulation in PC (Zhang et al., 2016; Zhang et al., 2020; Zhao et al., 2020), and an understanding of this network may provide important insights into the mechanisms behind PC tumorigenesis.

In this study, we explored genomic alterations in PC samples from the Gene Expression Omnibus (GEO) and The Cancer Genome Atlas (TCGA) cohorts and assessed patterns of RNA modifications. We found that RNA modification patterns were not only associated with infiltration of multiple immune cell types and clinical features, but also with AR pathway activation. Next, based on differentially expressed genes (DEGs) in the RNA modification pattern, we developed an RNA modification score (RM_Score) model of “writers” to quantify the efficacy of “writers” in individual patients. Finally, we assessed its therapeutic value in targeted therapies and immunotherapy and constructed a RM_Score-based CeRNA network.

## Methods

### Prostate cancer datasets source and preprocessing

The workflow of our study was shown in Supplementary Figure S1. Public gene-expression date and full clinical annotation were searched in the Cancer Genome Atlas (TCGA) database and Gene Expression Omnibus (GEO). Patients without survival information were removed from further evaluation. In total, two eligible PC cohorts (GSE70770, GSE116918) AND TCGA-PRAD (The Cancer Genome Atlas- Prostate Adenocarcinoma) were gathered in this study for further analysis. For microarray data, the normalized matrix files were directly downloaded. As to datasets in TCGA, RNA sequencing data (FPKM value) of gene expression and sample CNV information for prostate cancer samples were downloaded from the UCSC xena database (https://xenabrowser.net/datapages/), clinical information was downloaded using the R package cgdsr (version: 1.3.0), and mutation data was downloaded using the R package TCGAbiolinks (version: 2.16.4). It was then merged with the GSE70770 and GSE116918 chip expression data and batch effects from non-biological technical biases were corrected using the “ComBat” algorithm of sva package. (Version: 3.36.0) (Leek et al., 2012). In addition, copy number variation information for other tumors was downloaded using the R package TCGAmutations (version: 0.3.0).

The immunotherapy dataset for bladder cancer was downloaded using the R package IMvigor210CoreBiologies (version: 1.0.0)^15^. AS to chemotherapy datasets for breast cancer and advanced urothelial tumors (GSE25066 and GSE111636), the normalized matrix files were directly downloaded from GEO.

### Clustering expression pattern of 26 RNA modification “writers”

Cluster analysis of RNA-modified “writers” in 984 prostate cancer samples was performed using an unsupervised clustering algorithm. 7 m6A modification enzymes (METTL3, METTL14, WTAP, RBM15, RBM15B, ZC3H13, and KIAA1429), 4 m1A modification enzymes (TRMT61A, TRMT61B, TRMT10C, and TRMT6), 12 APA modification enzymes (CPSF1-4, CSTF1/2/3, PCF11, CFI, CLP1, NUDT21, and PABPN1), and 3 A- (ADAR, ADARB1, and ADARB2). Unsupervised clustering was used to identify robust prostate cancer clustering (Hartigan et al., 1979). For the preceding steps, we utilized the Consensus-Clusterplus package (version 1.52.0) (Wilkerson and Hayes, 2010) and conducted 1,000 repetitions to ensure the classification’s stability.

### Identification of differentially expressed genes (DEGs) between RNA modification distinct phenotypes

To identify RNA modification “writers”-related genes, we classified patients into two distinct m6A modification patterns based on the expression of 26 RNA modification “writers”. The empirical Bayesian approach of limma R package was applied to determine DEGs between different modification patterns. The criteria for determining DEGs was set as adjusted *p*-value <0.05 and |logFC|>0.58.

### Gene set variation analysis (GSVA) and estimation of TME cell infiltration

To study the differences of RNA modification patterns in biological processes, we used “GSVA” R package (version: 1.36.3) to conduct GSVA enrichment analysis (Hänzelmann et al., 2013). The gene set “c2. cp.kegg.v7.4” and “h.all.v7.4” for GSVA analysis was downloaded from the MSigDB database (https://www.gsea-msigdb.org/gsea/index.jsp, V7.4). The clusterProfiler R Package was used to functionally annotate 26 RNA modification enzyme genes (Yu et al., 2012).

To assess the proportion of 28 immune cell species in different subpopulations (data source: https://www.cell.com/cms/10.1016/j.celrep.2016.12.019/attachment/f353dac9-4bf5-4a52-bb9a-775e74d5e968/mmc3. xlsx), we obtained the degree of infiltration of 28 immune cell species using the ssGSEA (single sample gene set enrichment analysis) analysis in the R package GSVA (Charoentong et al., 2017).

### Generation of RM_Score

To quantify the RNA modification patterns of individual tumor, we constructed a set of scoring system to evaluate the RNA modification pattern of individual patients with prostate cancer—the RM_Score (Zeng et al., 2019). The procedures for establishment of RM_Score were as follows:

First, the DEGs identified from distinct RNA modification clusters were normalized across all PC samples, and the overlap genes were extracted. Using an unsupervised clustering method for analyzing overlap DEGs, the patients were divided into multiple groups for further examination. The consensus clustering algorithm was used to determine the number and stability of gene clusters. Then, using the univariate Cox regression model, we performed prognostic analysis on each gene in the signature. The significant prognostic genes were isolated for further analysis. Then, using principal component analysis (PCA), we constructed a gene signature relevant to m6A. Components 1 and 2 were both chosen to serve as signature scores.

### Calculation of TME cell invasion abundance

To quantify the relative abundance of 22 types of immune cells in colorectal cancer, we used CIBERSORT algorithm (https://cibersort.stanford.edu/) (Becht et al., 2016): the input mixture matrix is our gene expression matrix, the input is a gene signature reference for 22 immune cell types from Newman et al. (Newman et al., 2015), 100 times for permutation test, and RNA-seq data without quantile normalization, whereas microarray data with quantile normalization.

### Correlation between RM_Score and other related biological processes

In a study by Mariathasan et al., they constructed a set of gene sets that primarily contained genes associated with biological processes such as 1) immune-checkpoint; 2) antigen processing machinery; 3) CD8 T-effector signature; 4) epithelial-mesenchymal transition (EMT) markers including EMT1, EMT2 and EMT3; 5) Angiogenesis signature; 7) pan-fibroblast TGFb response signature (Pan-F-TBRS); 8) WNT targets; 9) DNA damage repair; 10) mismatch repair; 11) Nucleotide excision repair; 12) DNA replication; 13) Antigen processing and presentation. We quantified these biological functions in each sample using GSVA analysis to calculate an Enrichment score (ES), which further revealed links between samples with high and low RM_Score groupings and a few relevant biological pathways (Mariathasan et al., 2018).

### Association analysis of RM_Score and stromal score, immune score, estimate score, tumor purity

Immune scores, stromal scores, and tumor purity were calculated by R package estimate (version 1.0.13) based on specific gene expression profiles of immune and stromal cells by entering the gene expression profiles of the samples.

### Association analysis of RM_Score and drug sensitivity

Approximately 1,000 transcription profiles for cancer cell lines were obtained from Genomics of Drug Sensitivity in Cancer (GDSC) (Yang et al., 2013), available at (http://www.cancerrxgene.org/downloads) (Adams et al., 2007). We calculated the correlation between drug sensitivity and RM_Score using Spearman correlation analysis, where |Rs| > 0.35 and *p* 0.05 were considered significant correlations.

### ceRNA regulatory network construction

Differential miRNA, lncRNA, mRNA between RM_Score high and low risk groupings were identified by R package limma, where mRNA, miRNA, lncRNA screening criteria are |logFC|>0.58, *p* < 0.05, and miRNA-miRNA relationship pairs were downloaded from the miRTarBase database (http://mirtarbase.mbc.nctu.edu.tw/php/index.php), miRDB database and TargetScan database (http://www.targetscan.org/vert_72/)to download miRNA-miRNA targeting relationships, and then screened for mRNA_miRNA relationship pairs that were included in at least two sets of databases.

lncRNA-miRNA targeting relationships were downloaded from the TargetScan database (http://www.targetscan.org/vert_72/) to identify lncRNAs that have interactions with the above screened miRNAs. mRNA-miRNA-lncRNA networks were constructed by Cytoscape.

### Statistical analysis

Spearman and distance correlation were used to calculate the RNA modification “writers” expression correlation coefficient. The Wilcoxon test was utilized to evaluate the differences. Utilizing the receiver operating characteristic (ROC) curve, the model’s validity was determined.

On the basis of the correlation between RM Score and patient survival, the servicer package was utilized to establish the survival information cutoff point for each dataset. To reduce the calculated batch effect, the “surv-cutpoint” function was used to dichotomize RM Score, and all potential cutting points were repeatedly tested to determine the maximum rank statistic. Patients were then divided into the RM Score-high group and the RM Score-low group based on the maximum selected log-rank statistic. The Kaplan-Meier method was used to generate survival curves for prognostic analysis, and the log-rank test was applied to determine the significance of the differences. Utilizing a univariate Cox regression model, the hazard ratio (HR) between differentially expressed genes and “writers” was calculated. To determine whether RM Score is an independent predictor, we perform a multivariate Cox regression model analysis with age, gender, and stage as independent variables. All statistical analyses were two-sided, and *p* 0.05 was considered statistically significant.

## Results

### Landscape of genetic variation of 26 RNA modification “writers” in prostate cancer

The current study included 26 RNA modification “writers” (Supplementary Table S1), including three A-I modification “writers” seven m6A modification “writers” four m1A modification “writers” and twelve APA modification “writers."

**FIGURE 1:**
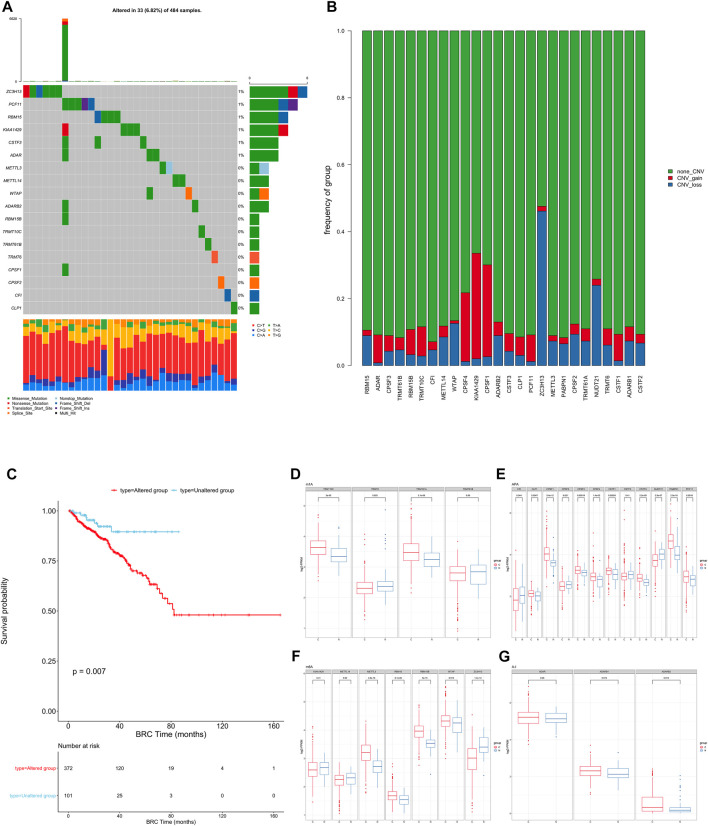
**(A)** The mutation frequency of RNA modification “writers” in 484 patients from the TCGA cohort. Each column represents individual patients. The upper bar graph shows TMB; the number on the right indicates the mutation frequency in each “writer”. The right bar graph shows the proportion of each variant type. The stacked bar graph below shows the fraction of conversions in each sample. **(B)** Bar graphs showing the frequency of CNV gain (red), loss (blue) and non CNV (green) of RNA modification “writers” in the TCGA-PRAD cohort. The height of each bar represents the alteration frequency. **(C)** Kaplan-Meier curves show overall survival of patients with (red) or without (blue) mutations in RNA modification “writers” in the TCGA-PRAD cohort. The grouping of PC samples is indicated at the bottom of the chart. *P* < 0.05 in the two-sided log-rank test was considered statistically significant. **(D–G)**. Box plots show the expression distribution of 26 “writers” of 4 types of RNA modification between paired normal (blue) and cancer (red) tissues. The boxes indicate the median ±1 quartile, with the whiskers extending from the hinge to the smallest or largest value within 1.5× IQR from the box boundaries.

To determine the genetic changes in RNA modification writers in pan-cancer, the prevalence of non-silent somatic mutations in 26 writers was evaluated. The mutation frequency of RNA writers was relatively low in the PRAD, PCPG, and UVM cohorts of the TCGA, while it was relatively high in the COAD cohort (Supplementary Figure S2A). Only 33 (29.46%) of the 484 PRAD samples contained mutations of RNA modification “writers” (Figure 1A). ZC3H13 had the highest mutation frequency (1%), followed by PCF11 and RBM15, whereas PABPN1 and NUDT21 did not exhibit any mutations in PRAD samples. PRAD patients with mutations of these “writers” showed a trend of longer overall survival rate than those without mutations though the difference showed non-statistically significant (Supplementary Figure S2B). Enrichment analysis’ Gene Set Variation Analysis (GSVA) was used to compare the signature gene sets of the “writers” mutation group and the non-mutation group. Myogenesis, Inflammatory response, and other pathways are upregulated in the mutation group, whereas MYC target and androgen response are downregulated. (Supplementary Figure S2D).

We then examined somatic copy number variation (CNV) of these writers in prostate cancer and found that KIAA1429 and CPSF1 had a widespread frequency of copy number variation (CNV) gain (Figure 1B). We defined patients with CNV or SNP as the mutation group and the rest of the samples as the non-mutation group, then went for further survival analysis. The overall survival of the mutant group was significantly lower than that of the non-mutant group. (Figure 1C).

To determine whether these genetic variations influenced the expression of RNA writers in PC patients, we compared the mRNA changes of regulators between paired normal and PC samples and found that the expression of most RNA writers was significantly elevated in PC (Figures 1D–G). Additionally, the analysis revealed that RNA authors with CNV gain were expressed at a higher level in cancer tissues (Figures 1D–G). RNA modification “writers” with CNV gain (e.g., CPSF1 and TRMT10C) were significantly more prevalent in PC tissues than in normal prostate tissue, indicating that CNV may be a regulator factor for “writer” mRNA expression. However, a subset of “writer” cells exhibited upregulated mRNA expression and a high frequency of CNV loss. To investigate the discrepancy between CNV values and mRNA expression in tumor samples, we divided the PC cohort into four groups based on their CNV values, which included CNV gain, CNV loss, and non-significant CNV alteration. Then, we compared the “writer” mRNA expression between these groups (Supplementary Figure S2C). In fact, patients with CNV gain exhibited higher expression levels than patients with CNV loss in these “writers.” CNV changes could not fully explain the differential expression of “writers” between tumor and normal tissues, as tumorigenesis is a complex process. Although many of the detected expression changes of “writers” can be explained by copy number variants (CNVs), CNVs are not the only factor that regulates mRNA expression. In addition to DNA methylation and transcription factors, additional factors can regulate gene expression.

This suggests that the mutation of “writer” including CNV and SNP, has potential role in the tumorigenesis and development of PC.

### Distinct patterns of RNA modification “writers” associated with cancer hallmarks and immune infiltration

A total of 948 prostate cancer samples and 125 control samples were selected for further analysis from three databases (TCGA, GSE116918, GSE70770) to obtain a more comprehensive understanding of the expression patterns of writers involved in tumorigenesis in prostate cancer.

**FIGURE 2:**
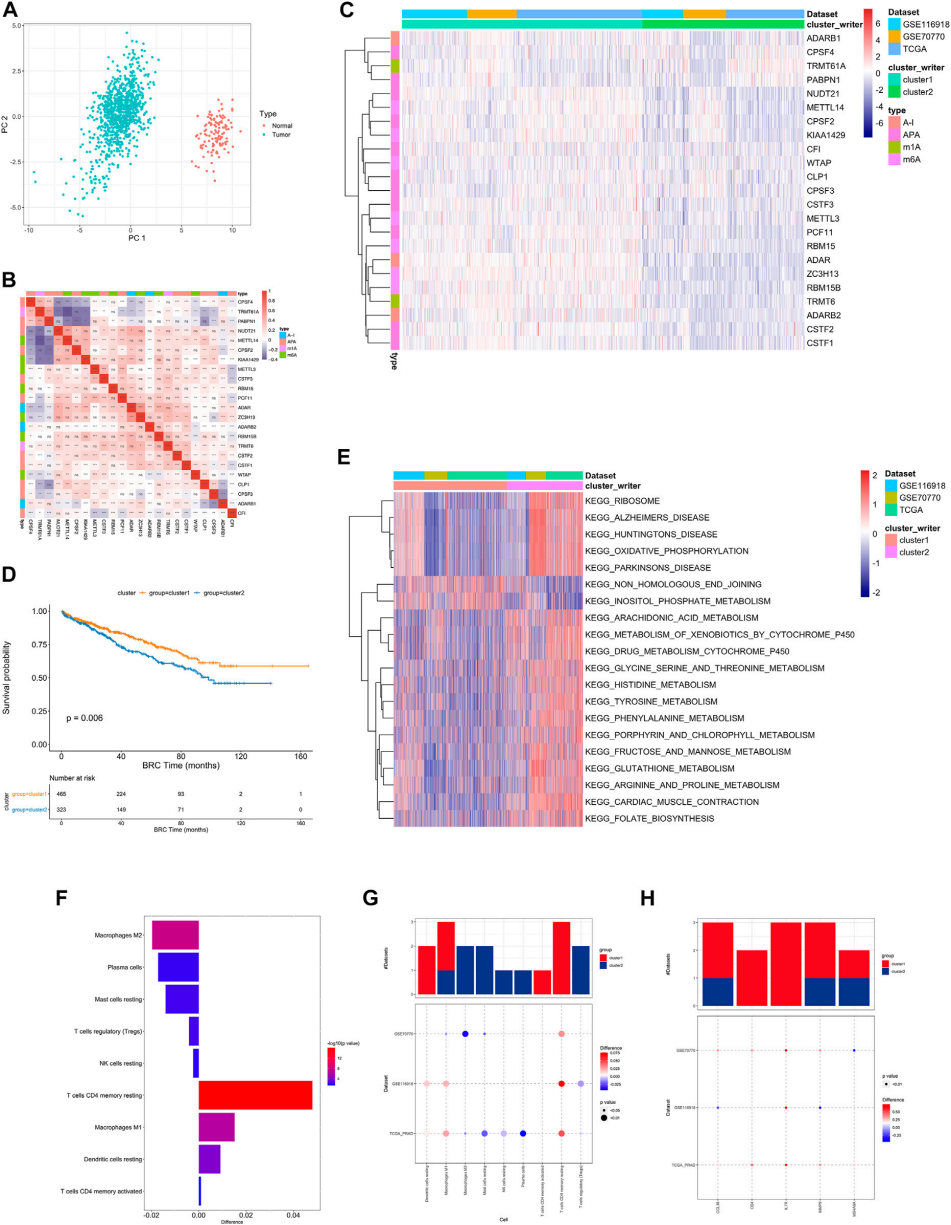
**(A)** PCA analysis of RNA writers between normal (red dot) and tumor (green) samples. **(B)** The difference in the relative abundance of immune cell infiltration in TME between RNA modification Cluster_1 and RNA modification Cluster_2 was calculated by the CIBERSORT algorithm. Difference >0 indicates that the immune cells were enriched in RNA modification Cluster_1, and the column color represents the statistical significance of the difference. **(C)** Heatmap shows a positive (red) and negative (blue) correlation among RNA modification “writers” in PC. *p < 0.05, **p < 0.01, and ***p < 0.001, as determined by the Spearman correlation analysis. **(D)** Kaplan-Meier curves compare overall survival between two RNA modification patterns, Cluster_1 (red) and Cluster_2 (blue), in all patients. The grouping of samples is shown at the bottom of the chart. *P* < 0.05 in the two-sided log-rank test was considered statistically significant. **(E)** Unsupervised clustering of 26 RNA modification “writers”. The clusters of PC cohorts and RNA modification types were used as sample annotations. Red, high expression of “writers”; blue, low expression. **(F)** Heatmap visualizing the GSVA enrichment analysis shows the activation states of biological pathways in distinct RNA modification patterns. Red, activated pathways; blue, inhibited pathways. The names of PC cohorts were used as sample annotations. **(G,H)**. The difference of immune cell infiltration **(G)** and expression of macrophage M2 and T cells CD4 memory resting marker genes **(H)** between RNA modification patterns. The upper bar graph shows the number of datasets that differ significantly between Cluster_1 and Cluster_2. The color of the bubble below the graph indicates the difference in each of the distinct GEO datasets, and 0the bubble size indicates the statistical significance of the difference. Difference >0 indicates that the infiltration of immune cells **(G)** or expression of macrophage M2 and T cells CD4 memory resting marker genes **(H)** were higher in RNA modification Cluster 1.

First, the distribution of the studied RNA writers in the genes were showed (Supplementary Figure S3A, Supplementary Table S7). PCA analysis of cancer and normal samples using these genes can clearly distinguish cancer samples from healthy control samples (Figure 2A).

Univariate Cox regression was performed on RNA writers and the samples were divided into two categories based on median gene expression values. 6 RNA writers were found to be associated with prostate cancer prognosis, including CPSF3, CSTF1, etc. (Supplementary Figure S1D, Supplementary Table S8).

A pairwise correlation was calculated between the expression of 26 writers in PC, and positive correlations were more common than negative correlations (Figure 2B). There was a significant correlation not only between the expression of RNA modification “writers” in the same category, but also among different types of modification writers.

In addition, we also performed consistent clustering of RNA writers expression profiles and presented the results in a network plot, which is shown in Supplementary Figure S3C The genes with black dots were positively correlated with prognosis and those with green dots were negatively correlated with prognosis, and the RNA writers gene regulatory network in this figure depicts the correlation between these genes interactions and the regulators with prognosis (Supplementary Table S9).

Based on the expression profiles of 23 selected RNA modification “writers” (Supplementary Table S12), we classified patients with qualitatively different RNA modification patterns using Consensus Clustering. According to unsupervised clustering, 567 patients from the combined datasets were assigned to Cluster_1, whereas 381 patients were assigned to Cluster_2 (Figure 2C). A prognostic analysis of RNA modification patterns revealed that Cluster_1 showed a marked survival advantage (Figure 2D, log-rank test, *p* = 0.006). An analysis of GSVA enrichment (Supplementary Table S13) was performed to determine the biological significance of these distinct RNA modification patterns. Cluster_1 was enriched in steroid hormone biosynthesis and prostate cancer pathways. Cluster_2 was enriched in ribosome, oxidative phosphorylation, and drug metabolism cytochrome P450 (Figure 2E).

Infiltrating immune cells from TMEs have been linked to RNA modification in numerous studies. Thus, we investigated the function of “writers” in TME. To determine the type of immune cells found in tumors, we used the CIBERSORT deconvolution algorithm, based on support vector regression, to compare immune cell types among RNA modification patterns (Supplementary Table S10). A significant correlation between CFI expression and dendritic cell resting was found, while a significant correlation between CPSF3 expression and plasma cells was found (Supplementary Figure S3B).

There were also significant differences in TME cell infiltration between the two RNA modification clusters (Figure 2F). We observed that Macrophages M1 and resting DCs were significantly higher in Cluster_1, whereas M2 macrophages, TREGs, and NK cells were significantly higher in Cluster_2 (Figures 2G,H). Accordingly, a comparison of the expression of macrophage markers in Cluster_1 and Cluster_2 indicated that M2 macrophage marker genes and T cell CD4 memory resting genes were significantly upregulated in Cluster_1 (Figure 2H). As a result, RNA modification patterns affected the degree of infiltration by certain immune cell types but did not alter the types of immune cells infiltrating the cells.

### Construction of RNA modification “writer” signature

**FIGURE 3:**
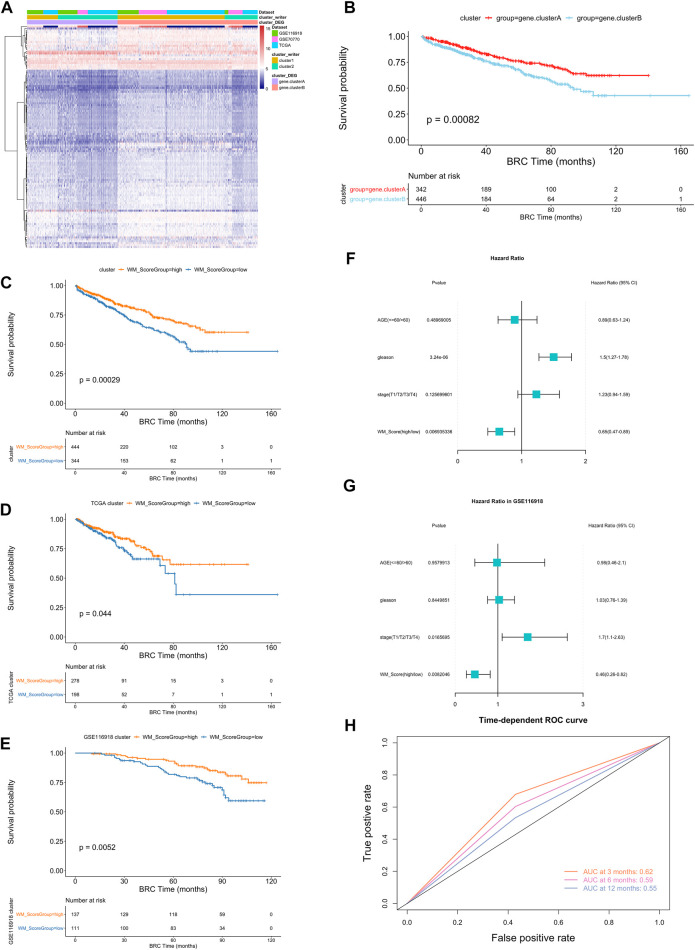
**(A)** Unsupervised clustering of the RNA modification phenotype-related genes. The names of those 3 PC cohorts were used as sample annotations. Red, high expression of phenotype-related genes; blue, low expression. **(B)** Kaplan-Meier curves comparing overall survival between two DEG clusters, gene. cluster_A (red) and gene. cluster_B (blue), in the 3 PC cohort. The grouping of PC samples is shown under the Kaplan-Meier plot. *P* < 0.05 in the two-sided log-rank test was considered statistically significant. **(C–E)** Kaplan-Meier curves show overall survival in WM_Score-high (red) and -low (blue) in all samples **(C)**, TCGA **(D)** and GSE116918 **(E)**. The grouping of PC samples is shown at the bottom of the chart. *P* < 0.05 in the two-sided log-rank test was considered statistically significant. **(F–G)**. Multivariate Cox regression model analysis, which included the factors of WM_Score, patient age, Gleason score, TNM status, and patient outcomes in the overall samples **(F)** and GSE116918 **(G)** cohorts. The length of the horizontal line represents the 95% confidence interval (CI) for each group. The vertical dotted line represents the hazard ratio (HR) of all patients shown by the forest plot. **(H)** The predictive value of WM_Score in patients (AUC:0.62, 0.59, and 0.55, 3, 6, 12-month overall survival).

To further analysis those two RNA modification patterns in the prostate cancer, we identified 116 RNA modification related differential expressed genes and performed enrichment analysis (Figure 3A, Supplementary Table S15, screening criteria were |logFC|>0.58, *p*-value < 0.05). These differential genes were mainly associated with some metabolic pathways (Supplementary Table S17). To further validate this differential regulation, an unsupervised clustering analysis was performed on these differential genes. This analysis classified patients into two genetic subtypes: gene cluster A and gene cluster B (Supplementary Table S16), and the two subtype clusters showed significant differences in prognosis (Figure 3B).

In order to quantify the RNA modification pattern of individual patients with PC, we constructed a DEGs-based score model based on these phenotype-related genes; this model was referred to as the RM_Score (“Writers” of RNA Modification_Score; see Methods). According to the surv cut point function in the R package survminer, the best threshold points for RM_Score classification was determined (cutoff = 0.0212,004) and the samples were classified into two categories, RM_Score high and RM_Score low (Supplementary Table S19), the RM_Score high and RM_Score low samples were significantly different in prognosis across all samples, RM_Score high (Figure 3C). The similar results are observed in the TCGA (Figure 3D) and GSE116918 (Figure 3E) datasets. And the area under the ROC curve for prediction of survival at 3, 6 and 12 months reached 0.62, 0.59 and 0.55 (Figure 3H). The subtypes obtained from the second clustering analysis also had significantly different RM_Score in the two previous clustering analyses. Based on Wayne diagrams and histograms of frequency distributions (Supplementary Figure S4A–D), A comparison of these three classifications revealed that the latter two classifications were calculated consistently (Supplementary Table S20).

### Molecular subtypes and clinical characteristics associated with RM_Score in PC

**FIGURE 4:**
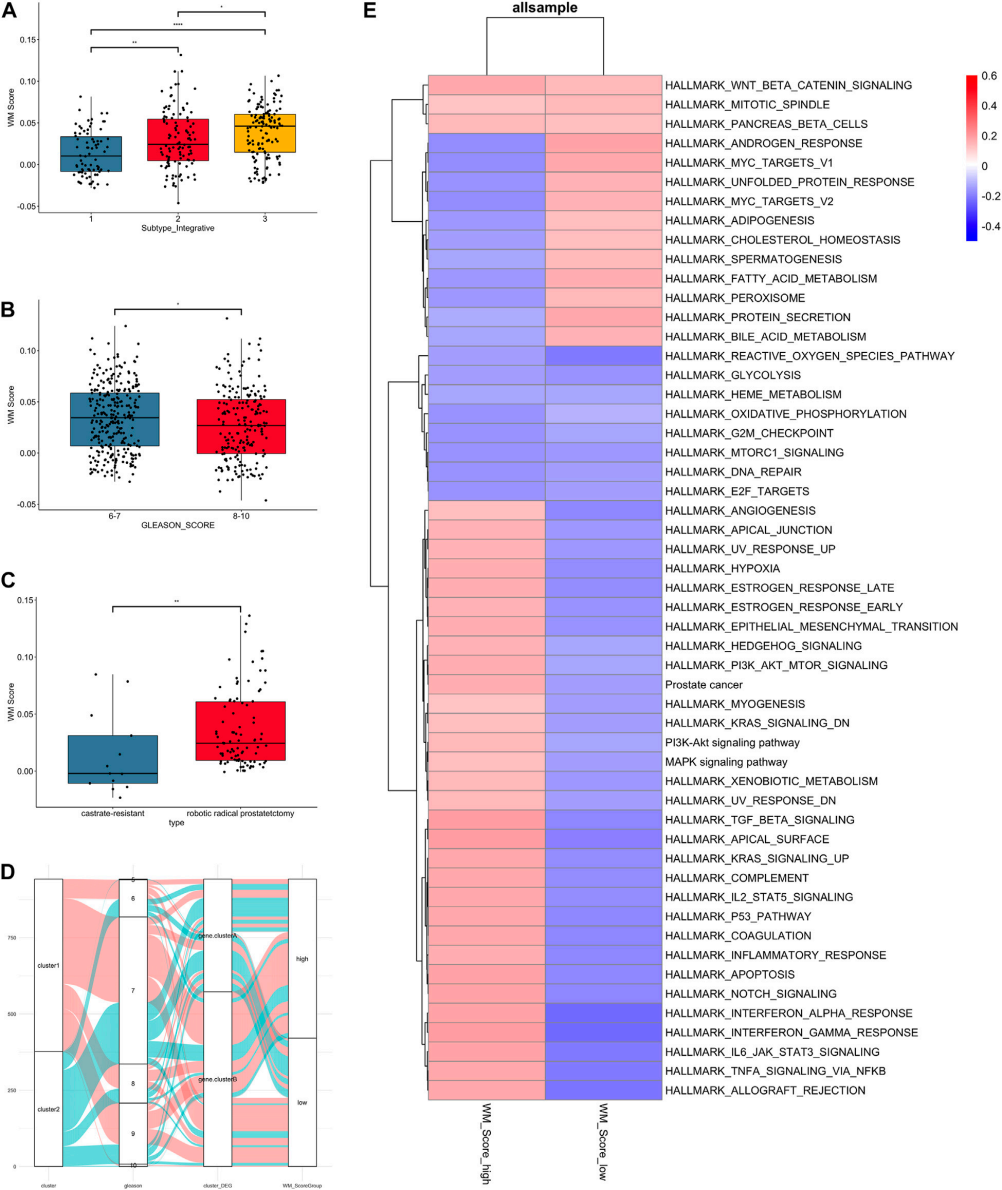
Clinical and biological characteristics of PC associated with the WM_Score. **(A)** WM_Score differences among TCGA molecular subtypes of PC in TCGA-RAD datasets. **(B)** Differences in the WM_Score between Gleason score 6-7 (blue) and 8-10 (red) group in the 3 PC cohort. **(C)** Differences in the WM_Score between castrate-resistant (blue) and robotic radical prostatectomy (red) group in the GSE70770 cohort. Wilcoxon test was used to assess the difference. The boxes indicate the median ±1 quartile, with the whiskers extending from the hinge to the smallest or largest value within 1.5× IQR from the box boundaries. **(D)** The Sankey diagram shows the variation in the distribution of samples across these classifications **(E)** Heatmap shows the differences in enrichment in the characteristic signaling pathways of PC subtypes between WM_Score-high and -low groups in all samples. Red, high enrichment score; blue, low enrichment score.

An analysis of multivariate Cox regression using the patient’s clinical characteristics, including age, Gleason score and stage status, was conducted to determine whether the RM_Score could be used as an independent prognostic factor (Figure 3F). The analysis showed that both Gleason and RM_Score were significantly associated with prognosis in the full sample (Figures 4A,B, Supplementary Table S22). A sample of prostate cancer patients from the GSE116918 database was used to validate the reliability of the RM_Score, in which the data set demonstrated a significant correlation between the stage and the RM_Score. (Figure 3G, Supplementary Table S22).

The result of comparison between different subtype and Gleason score in all patients were shown (Figures 4A,B). Also, we compared the variability of RM_score between hormone resistant and sensitive samples in the GSE70770 set and it showed a significant difference in scores between two (Figure 4C). Notably, lower RM_score was found in higher Gleason score group and castration resistant group, which corresponded to the result of the survival analysis in the previous section, i.e., the low RM_score group had a poor prognosis. It is not absolute, however. Figure 4D illustrates the flow of various Gleason scores between RM_score groups for all datasets. Some low Gleason score patients are categorized as low-RM_score groups, whilst some high Gleason score patients are categorized as high RM_score groups.

As to the GSVA analysis, most pathways were up-regulated in RM_score high group, like PI3K, KRAS, MAPL, P53, estrogen response pathway, while androgen response and MYC targets were up-regulated in RM_score low group (Figure 4E).

Using the maftools package, we then analyzed the distribution differences of somatic mutation between low and high RM score in the TCGA-PRAD cohort. As shown in Supplementary Figure S7A,B, the low RM score group had a mutation burden comparable to the high RM score group. The high frequency of mutational load suggests that these genes play a significant role in the development of prostate cancer, despite the absence of a statistically significant difference between the two groups.

### Analysis of the relationship between RM_Score and immune infiltration

**FIGURE 5:**
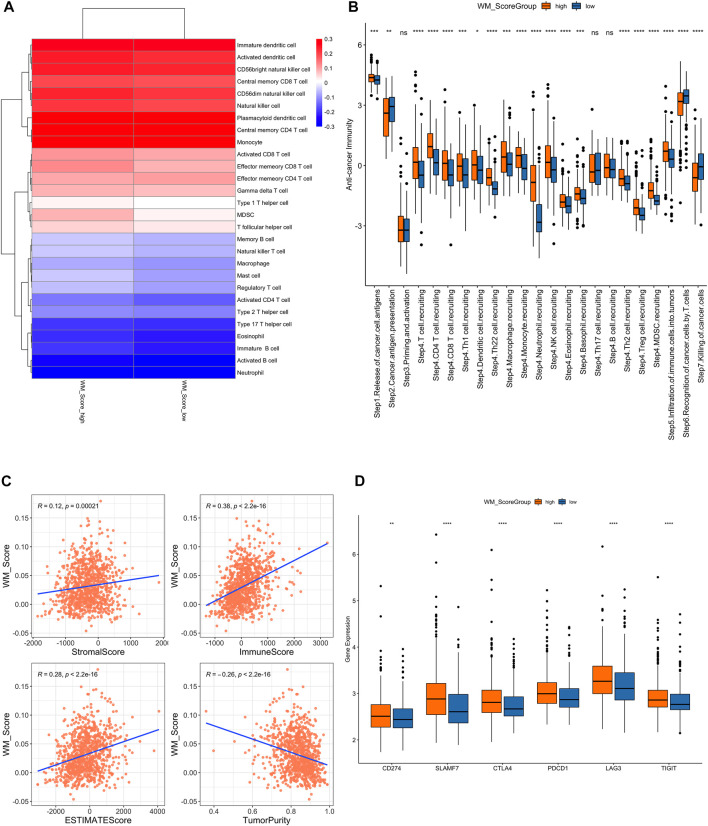
**(A)**. Difference of immune cell infiltration in the WM_Score-high/-low. **(B)** Analysis of the difference in anti-tumor immune process activity between high and low risk groups with WM_Score scores. **(C)** Correlation of WM_Score scores with stromal score, immune score, and tumor purity (A: stromal score; **(B)** immune score; **(C)** estimate score; **(D)** tumor purity) by Spearman analysis. **(D)** Gene expression of immune checkpoint expression in WM_Score -high and -low.

We used ssGSEA to analyze the differences in immune infiltrating cell types between the RM_Score high and low risk groups (Supplementary Table S25). The results are shown in Figure 5A. However, there is no significant difference in all cell types between RM_score high and low groups, which indicates RNA modification patterns may affect the degree of infiltration but did not alter the types of infiltrating immune cells.

We also performed a correlation analysis between RM_Score and stromal score, immune score, and tumor purity (Figure 5C, Supplementary Table S26). The stromal and immune score were proportional to RM_score but tumor purity had negative correlation to RM_score.

We downloaded the results of the TIP analysis of TCGA prostate cancer samples from the TIP (Tracking Tumor Immunophenotype) website (http://biocc.hrbmu.edu.cn/TIP/) and then compared the differences in anti-tumor immune process activity between the high and low RM_Score risk groups. We also analyzed the differential expression of immune checkpoint expression in those two groups. The RM_Score high group showed significantly higher expression levels than the RM_Score low group, as shown in Figure 5B, importantly in step4 T cell recruiting, while the step7 killing of cancer cells are more active in RM_score low group.

Additionally, we examined the differences in immune checkpoint expression between the WM Score high and low score groups. These genes’ expression levels varied significantly between the two groups of samples, and they were significantly higher in the WM Score high group than in the WM Score low group (Figure 5D).

### The role of RM_Score in drug sensitivity and immunotherapy and chemotherapy efficacy

**FIGURE 6:**
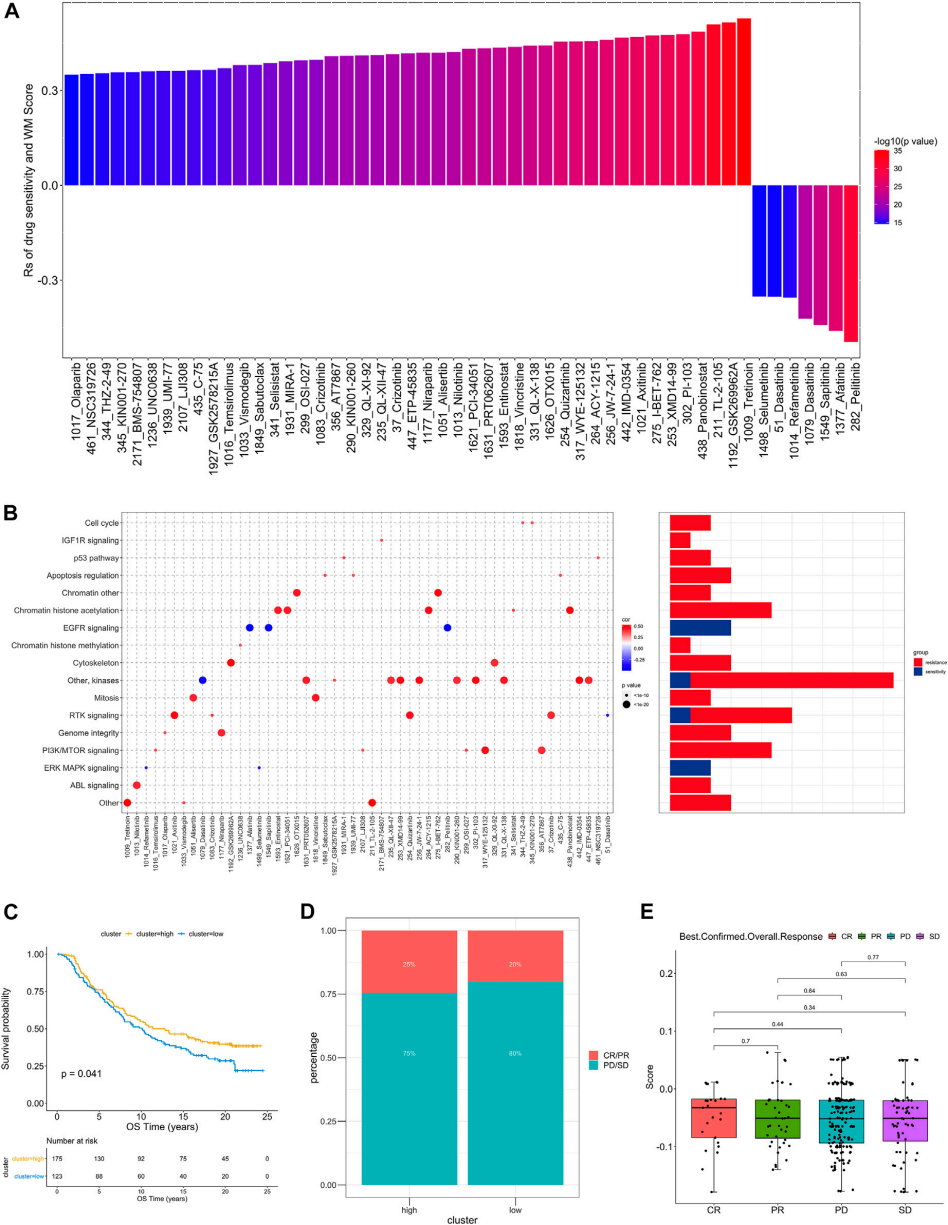
The relationship between WM_Score and drug sensitivity and efficacy of immunotherapy. **(A)** The correlation between WM_Score and drug sensitivity evaluated by the Spearman analysis. Each column represents a drug. The color of the column indicates the significance of the correlation. The height of the column indicates the correlation, indicates that WM_Score related to drug resistance (Rs > 0) or drug sensitive (Rs < 0) to WM_Score. **(B)** Signaling pathways targeted by drugs that are resistant (red) or sensitivity (blue) to the WM_Score. Drug names are listed on the horizontal axis and the signaling pathway targeted by the drug on the vertical axis. The bar graph on the right shows the number of drugs targeting each signaling pathway. The size of the point indicates the significance of the correlation. **(C)** Kaplan-Meier curves show overall survival in the WM_Score-high (red) and -low (blue) subgroups after the immunotherapy in the IMvigor210 cohort. The grouping of patients is shown at the bottom of the chart. *P* < 0.05 in the two-sided log-rank test was considered statistically significant. **(D)** The proportion of patients in the IMvigor210 cohort with different responses to PD-L1 blockade immunotherapy. The fisher test was used to determine the statistical significance of the difference. SD, stable disease; PD, progressive disease; CR, complete response; PR, partial response. **(E)** The difference in the WM_Score between distinct clinical outcomes of anti-PD-L1 treatment in the IMvigor210 cohort.

In order to further understand how the RM_Score affects drug response, we assessed the relationship between the RM_Score and the response to drugs in cancer cell lines. As a result of Spearman correlation analysis, we identified 52 significant correlations between RM_Score and drug sensitivity in the Genomics of Drug Sensitivity in Cancer (GDSC) database (Figure 6A, Supplementary Table S28). Among them, forty-five pairs showed that drug sensitivity correlated with the RM_Score, including the EGFR inhibitor Afatinib (Rs = − 0.26, *p* = 4.12 × 10–13). Seven pairs exhibited drug resistance correlated with the RM_Score, including PARP inhibitor Olaparib (Rs = 0.35 *p* = 2.60E-15), TKI inhibitor Axitinib (Rs = 0.47, *p* = 1.04E-27). A further analysis was conducted to examine the signaling pathways of the genes targeted by these drugs. It was found that drugs whose sensitivity was associated with high RM_Score targeted cell cycle, chromatin, and RTK signaling pathways. In contrast, the drugs associated with low RM Score sensitivity targeted the MAPK and EGFR pathways (Figure 6B). These findings suggest a correlation between RNA modification patterns and drug sensitivity. Thus, the RM Score may serve as a biomarker for determining the most effective treatment strategies.

**FIGURE 7:**
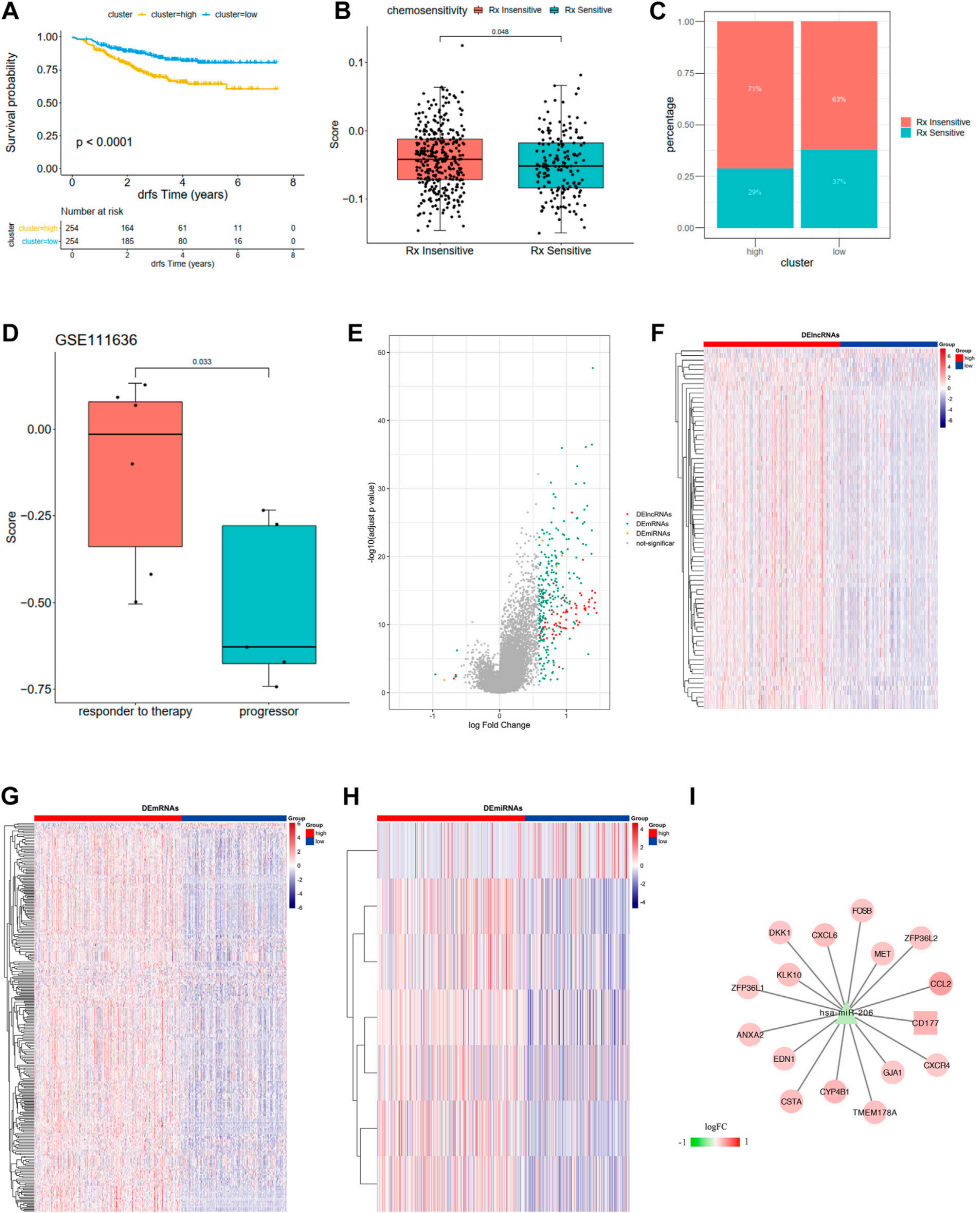
**(A)** Kaplan-Meier curves show overall survival in the WM_Score-high (yellow) and -low (blue) subgroups after the immunotherapy in the GSE25066. The grouping of patients is shown at the bottom of the chart. *P* < 0.05 in the two-sided log-rank test was considered statistically significant. **(B)** The proportion of patients in the GSE25066 with different responses to chemotherapy. The fisher test was used to determine the statistical significance of the difference. **(C)** The difference between distinct between the WM_Score high and low groups in the GSE25066. **(D)** The difference in the WM_Score between distinct clinical outcomes of anti-PD-1 treatment in the GSE111636. **(E–I)** WM_Score -High and -low differential expression of miRNA, lncRNA, mRNA (E: differential volcano map; FGH: differential lncRNA, mRNA, miRNA heat map, respectively) and ceRNA network (I: square is lncRNA, triangle is miRNA, circles are mRNAs).

As the RM_Score appears to be associated with the immune microenvironment of the tumor (Figure 5C), we examined its ability to predict the response of patients to ICB treatment. A total of two immunotherapy cohorts were analyzed in this study. As shown in Figure 6C, the RM_Score high group had significant clinical benefits and a markedly prolonged overall survival in the anti-PD-L1 cohort (IMvigor210). However, there was no significant difference between the 348 IMvigor210 patients’ responses to anti-PD-L1 blockers (Figures 6D,E), including complete responses (CR), partial responses (PR), stable diseases (SD), and progressive diseases (PD). As shown in Figure 6E, we didn’t observe significant differences between the immune subtypes of IMvigor210, namely “immune inflamed”, “immune excluded”, and “immune desert”. Additionally, TMB and neoantigen burden were similar in groups with low and high RM_Score (Supplementary Figures S4C–E). However, the situation is different in another anti-PD-1 cohort (GSE111636). The RM_score was significantly higher in responder to anti-PD-1 therapy group than progressor group (Figures 7C,D).

The situation is even more interesting in a breast cancer chemotherapy cohort when we tried to better define the role of RM_score in chemotherapy. Unlike the previous prostate cancer cohort and the high-grade uroepithelial cancer cohort (GSE111636), the survival analysis of the breast cancer cohort showed significantly better survival in the low group than in the high group (Figure 7A, *p* < 0.0001). Also, the chemotherapy sensitive group exhibited lower RM_score than insensitive group (Figure 7B, *p* = 0.048).

### RM_Score related ceRNA network construction

We then used R package limma to identify RM_Score high and low risk grouping differential miRNAs, lncRNAs, and mRNAs. mRNA, miRNA, and seven differential miRNAs, and 77 differential lncRNAs were screened, respectively (Figures 7E–H, Supplementary Table S30). Then the ceRNA network was constructed by combining the regulatory relationships of the database. The network contained a total of 15 mRNAs, 1 lncRNA and 1 miRNA (Figure 7I).

## Discussion

Increasing evidence suggests that RNA modifications play a crucial role in inflammation, innate immunity, and anti-tumor activity by interacting with a variety of “writers.” The interrelationships and functions of multiple types of RNA modification “writers” in cancer are not yet fully understood. Here, we reveal the overall variation in RNA modifications at the transcriptional level: m6A, m1A, APA, and A-to-I RNA editing enzymes; and their interrelationship in prostate cancer.

We identified two distinct RNA modification patterns based on 26 RNA modifying enzymes, defined two subtypes of prostate cancer associated with RNA modification, and developed a scoring model, RM Score, to evaluate the effectiveness of RNA modification “writers” in individual patients. Intriguingly, it was associated with a better prognosis in breast cancer but a worse prognosis in prostate and bladder cancer. In addition, the subtype with a high RM Score is distinguished by a significant inhibition of the AR signaling pathway and a significant activation of the estrogen pathway.

In our study, RM_score scores had opposite prognostic predictive effects for two different tumors, the urological cancer and breast cancer. It has been shown that the androgen pathway and estrogen pathway play a different role in these two types of tumors. Several key signaling pathways cross over with the AR pathway, including the PI3K/Akt/mTOR and MAPK pathways, as well as hormone receptors such as the estrogen receptor and human epidermal growth factor receptor 2.

It has been reported that androgens not only increase nAR-positive BCa cell infiltration *via* the classical nAR, but also that DHT and the novel membrane receptor mAR-SLC39A9 may increase migration and infiltration of nAR-negative BCa cells by altering Gαi protein-regulated MAPK/MMP9 intracellular signaling (Chen et al., 2020). Inoue et al. found that AR pathway is involved in the tumor growth modulating ATF2 activity through ERK in bladder cancer cells (Inoue et al., 2018).

Activated AR, on the other hand, inhibited the growth of breast cancers driven by the ER through displacement of the ER and critical transcriptional co-activators from chromatin, which resulted in transcriptional downregulation (Hickey et al., 2021).

RNA modifications and androgen pathway effects have been linked to prostate cancer progression in recent studies. The gradual decrease of METTL14 (methyltransferase like 14) and the increase of ALKBH5 affected the activity of AMPK, causing an inhibition of autophagy and a subsequent suppression of testosterone synthesis in Leydig cells (Chen et al., 2021b). According to other studies, SIAH1 is a tumor suppressor involved in PC pathogenesis by repressing CPSF1-mediated AR-v7 generation and is a key regulatory factor (Xia et al., 2022).

mi206 played a key role in the differentially expressed ceRNA network in high- and low-RM_Score subtypes, and mi206 expression has been reported to be significantly upregulated in the tumor-associated stromal fraction. Nevertheless, mi206 plays a different role in prostate cancer and breast cancer. It has been demonstrated that miR-206 is highly expressed in breast tumors with no estrogen receptor compared with those with estrogen receptor positive breast tumors (Kondo et al., 2008). Specific to ER-negative breast cancer, miR-206 expression is higher than in ER-positive breast cancer (Adams et al., 2007). MiR-206 induces estrogen non-dependent state in MCF-7 cells when forced to express in these cells. Moreover, mi206 is significantly upregulated in prostate cancer, but functions as a tumor suppressor (Goljanek-Whysall et al., 2012; Singh et al., 2013; Walter et al., 2013).

Finally, we demonstrate the potential therapeutic efficacy of RNA modifiers in prostate cancer. According to the RM_Score, resistance to drugs targeting the cell cycle or apoptotic pathways may be associated with resistance to drugs targeting MAPK or EGFR signaling pathways. These findings imply that patients with a higher RM Score may benefit more from drugs that target these signaling pathways than from those that target the cell cycle or apoptotic pathways. By identifying distinct RNA modifications in tumors, our findings expand the scope for personalized chemotherapy and targeted treatment of prostate cancer. Due to the retrospective nature of the cohort and all our results are derived from bioinformatics analyses of publicly available databases, further additional experimental research is required. Still, our findings are supported by multiple independent GEO datasets, demonstrating their validity. Second, based on the expression patterns of 24 writers to represent distinct RNA modification patterns, we divided PC patients into two clusters. Due to the lack of large-scale m6A-seq performed in the PC cohorts, the precise RNA modification landscape is unclear in clinical practice. Due to the lack of available data sets, we only validated a few data sets, including breast cancer and bladder cancer data sets. To draw more precise conclusions, this relationship must be investigated further in a large clinical cohort.

## Data Availability

The original contributions presented in the study are included in the article/Supplementary Material, further inquiries can be directed to the corresponding authors.

## Abbreviations

APA, Alternative polyadenylation; M1A, N1-methyladenosine; A-to-I, Adenosine-to-inosine; BCa, Breast cancer; CNV, Copy number variation; DEGs, Differentially expressed genes; GEO, Gene Expression Omnibus; GSVA, Gene set variation analysis; HR, Hazard ratios; ICB, Immunological checkpoint blockade; PC, Prostate cancer; m6A, N6-methyladenosine; PCA, Principal component analysis; ssGSEA, Single-sample gene-set enrichment analysis; TCGA, The Cancer Genome Atlas; TME, Tumor microenvironment.
