# Supplementary material for: RNA modification writers pattern in relation to tumor microenvironment and prognosis in prostate cancer
Source: Front Genet. 2023 Jan 18;13:1065424. doi: 10.3389/fgene.2022.1065424 (PMC9889935; doi:10.3389/fgene.2022.1065424)
Supplement: Supplementary file 2 [file DataSheet2.DOCX]

**Figure 1**

**A.** The mutation frequency of RNA modification “writers” in 484 patients from the TCGA cohort. Each column represents individual patients. The upper bar graph shows TMB; the number on the right indicates the mutation frequency in each ”writer”. The right bar graph shows the proportion of each variant type. The stacked bar graph below shows the fraction of conversions in each sample.

**B.** Bar graphs showing the frequency of CNV gain (red), loss (blue) and non CNV (green) of RNA modification “writers” in the TCGA-PRAD cohort. The height of each bar represents the alteration frequency.

**C.** Kaplan-Meier curves show overall survival of patients with (red) or without (blue) mutations in RNA modification “writers” in the TCGA-PRAD cohort. The grouping of PC samples is indicated at the bottom of the chart. p < 0.05 in the two-sided log-rank test was considered statistically significant.

**D-G.** Box plots show the expression distribution of 26 “writers” of 4 types of RNA modification between paired normal (blue) and cancer (red) tissues. The boxes indicate the median ± 1 quartile, with the whiskers extending from the hinge to the smallest or largest value within 1.5× IQR from the box boundaries.

**Figure 2**

**A.** PCA analysis of RNA writers between normal (red dot) and tumor (green) samples.

**B.** The difference in the relative abundance of immune cell infiltration in TME between RNA modification Cluster_1 and RNA modification Cluster_2 was calculated by the CIBERSORT algorithm. Difference > 0 indicates that the immune cells were enriched in RNA modification Cluster_1, and the column color represents the statistical significance of the difference.

**C.** Heatmap shows a positive (red) and negative (blue) correlation among RNA modification “writers” in PC. *p < 0.05, **p < 0.01, and ***p < 0.001, as determined by the Spearman correlation analysis.

**D.** Kaplan-Meier curves compare overall survival between two RNA modification patterns, Cluster_1 (red) and Cluster_2 (blue), in all patients. The grouping of samples is shown at the bottom of the chart. p < 0.05 in the two-sided log-rank test was considered statistically significant.

**E.** Unsupervised clustering of 26 RNA modification “writers” . The clusters of CRC cohorts and RNA modification type were used as sample annotations. Red, high expression of “writers” ; blue, low expression.

F. Heatmap visualizing the GSVA enrichment analysis shows the activation states of biological pathways in distinct RNA modification patterns. Red, activated pathways; blue, inhibited pathways. The names of PC cohorts were used as sample annotations.

**G-H.** The difference of immune cell infiltration (G) and expression of macrophage M2 and T cells CD4 memory resting marker genes (H) between RNA modification patterns. The upper bar graph shows the number of datasets that differ significantly between Cluster_1 and Cluster_2. The color of the bubble below the graph indicates the difference in each of the distinct GEO datasets, and the bubble size indicates the statistical significance of the difference. Difference > 0 indicates that the infiltration of immune cells (G) or expression of macrophage M2 and T cells CD4 memory resting marker genes (H) were higher in RNA modification Cluster 1.

**Figure 3**

**A.** Unsupervised clustering of the RNA modification phenotype-related genes. The names of those 3 PC cohorts were used as sample annotations. Red, high expression of phenotype-related genes; blue, low expression.

**B.** Kaplan-Meier curves comparing overall survival between two DEG clusters, gene.cluster_A (red) and gene.cluster_B (blue), in the 3 PC cohort. The grouping of PC samples is shown under the Kaplan-Meier plot. p < 0.05 in the two-sided log-rank test was considered statistically significant.

**C-E.** Kaplan-Meier curves show overall survival in WM_Score-high (red) and -low (blue) in all samples (C), TCGA (D) and GSE116918 (E). The grouping of PC samples is shown at the bottom of the chart. p < 0.05 in the two-sided log-rank test was considered statistically significant.

**F-G.** Multivariate Cox regression model analysis, which included the factors of WM_Score, patient age, Gleason score, TNM status, and patient outcomes in the overall samples (F) and GSE116918 (G) cohorts. The length of the horizontal line represents the 95% confidence interval (CI) for each group. The vertical dotted line represents the hazard ratio (HR) of all patients shown by the forest plot.

**H.** The predictive value of WM_Score in patients (AUC:0.62, 0.59, and 0.55, 3, 6, 12-months overall survival).

**Figure 4. Clinical and biological characteristics of PC associated with the WM_Score.**

**A.** WM_Score differences among TCGA molecular subtypes of PC in TCGA-RAD datasets.

**B.** Differences in the WM_Score between Gleason score 6-7 (blue) and 8-10 (red) group in the 3 PC cohort.

**C.** Differences in the WM_Score between castrate-resistant (blue) and robotic radical prostatectomy (red) group in the GSE70770 cohort. Wilcoxon test was used to assess the difference. The boxes indicate the median ± 1 quartile, with the whiskers extending from the hinge to the smallest or largest value within 1.5× IQR from the box boundaries.

**D.** The Sankey diagram shows the variation in the distribution of samples across these classifications

**E.** Heatmap shows the differences in enrichment in the characteristic signaling pathways of PC subtypes between WM_Score-high and -low groups in all samples. Red, high enrichment score; blue, low enrichment score.

**Figure 5**

**A.** Difference of immune cell infiltration in the WM_Score-high/-low.

**B.** Analysis of the difference in anti-tumor immune process activity between high and low risk groups with WM_Score scores.

**C.** Correlation of WM_Score scores with stromal score, immune score, and tumor purity (A: stromal score; B: immune score; C: estimate score; D: tumor purity) by Spearman analysis.

**D.** Gene expression of immune checkpoint expression in WM_Score -high and -low.

**Figure 6. The relationship between WM_Score and drug sensitivity and efficacy of immunotherapy.**

**A.** The correlation between WM_Score and drug sensitivity evaluated by the Spearman analysis. Each column represents a drug. The color of the column indicates the significance of the correlation. The height of the column indicates the correlation, indicates that WM_Score related to drug resistance (Rs > 0) or drug sensitive (Rs < 0) to WM_Score.

**B.** Signaling pathways targeted by drugs that are resistant (red) or sensitivity (blue) to the WM_Score. Drug names are listed on the horizontal axis and the signaling pathway targeted by the drug on the vertical axis. The bar graph on the right shows the number of drugs targeting each signaling pathway. The size of the point indicates the significance of the correlation.

**C.** Kaplan-Meier curves show overall survival in the WM_Score-high (red) and -low (blue) subgroups after the immunotherapy in the IMvigor210 cohort. The grouping of patients is shown at the bottom of the chart. p < 0.05 in the two-sided log-rank test was considered statistically significant.

**D.** The proportion of patients in the IMvigor210 cohort with different responses to PD-L1 blockade immunotherapy. The fisher test was used to determine the statistical significance of the difference. SD, stable disease; PD, progressive disease; CR, complete response; PR, partial response.

**E.** The difference in the WM_Score between distinct clinical outcomes of anti-PD-L1 treatment in the IMvigor210 cohort.

**Figure 7**

A. Kaplan-Meier curves show overall survival in the WM_Score-high (yellow) and -low (blue) subgroups after the immunotherapy in the GSE25066. The grouping of patients is shown at the bottom of the chart. p < 0.05 in the two-sided log-rank test was considered statistically significant.

B. The proportion of patients in the GSE25066 with different responses to chemotherapy. The fisher test was used to determine the statistical significance of the difference.

C. The difference between distinct between the WM_Score high and low groups in the GSE25066.

D. The difference in the WM_Score between distinct clinical outcomes of anti-PD-1 treatment in the GSE111636.

E-G. WM_Score -High and -low differential expression of miRNA, lncRNA, mRNA (E: differential volcano map; FGH: differential lncRNA, mRNA, miRNA heat map, respectively) and ceRNA network (square is lncRNA, triangle is miRNA , circles are mRNAs).

**Figure S1. Flowchart of the steps in the performed analyses.**

**Figure S2**

**A.** The mutation frequency of RNA modification “writers” among 33 cancer types in the TCGA cohort. The horizontal axis represents cancer types, and the number of samples is given in the parentheses. The vertical axis lists the names of the genes.

**B.** Kaplan-Meier curves show overall survival of patients with (red) or without (blue) somatic mutations in RNA modification “writer” in all patients. The grouping of PC samples is indicated at the bottom of the chart. p > 0.05 in the two-sided log-rank test was considered statistically non-significant.

**C.** The distribution of correlation coefficient between “writers” expression and CNV in all samples. |Rs| > 0.3 and p value < 0.05 indicates that “writers” expression is related to CNV. (C) The sample size for each group based on the CNV alteration (CNV loss/ CNV gain/ normal/ none CNV). Wilcoxon test was used to assess the difference. The boxes indicate the median ± 1 quartile, with the whiskers extending from the hinge to the smallest or largest value within 1.5× IQR from the box boundaries.

**D.** Comparison of GSEA enrichment analysis between “writers” mutation samples and non-mutation samples. NES, Normalized enrichment score

**Figure S3**

**A.** Position of RNA writers on chromosomes.

**B.** Heatmap shows the positive (red) and the negative (blue) correlation between TME infiltration and WM_Score in PC. *p < 0.05, **p < 0.01, and ***p < 0.001, as determined by the Spearman correlation analysis.

**C.** Interaction of RNA writers (green dots in the circles indicate prognostic protective factors, black dots in the circles indicate prognostic risk factors. The lines connecting the genes show their interactions, with negative correlations marked in blue and positive correlations in red)

**D.** Association of gene expression for 26 RNA modification “writers” with patient overall survival times based on Univariate Cox regression analysis in 3 cohort.

**E.** Kaplan-Meier curves show overall survival in WM_Score-high (red) and -low (blue) in GSE70770. The grouping of PC samples is shown at the bottom of the chart.

**Figure S4.**

**A-B.** Overlap (A) and frequency (B) of classifiers of WM_Score-high/−low and Cluster_1/2 in PC.

**C-D.** Overlap (C) and frequency (D) of classifiers of WM_Score-high/−low and gene.cluster_A/B in PC. The Fisher test was used to determine the statistical significance of the difference.

**E.** Heatmap shows the differences in enrichment in the characteristic signaling pathways of PC subtypes between WM_Score-high and -low groups in the TCGA cohort. Red, high enrichment score; blue, low enrichment score.

**Figure S5**

**A-B.** Molecular characterization of WM_Score risk groups (A: distribution of mutations in WM_Score high samples; B: distribution of mutations in WM_Score low samples)

**C.** The difference in the WM_Score among immune phenotypes, including the inflamed (green), excluded (blue), and desert (red) immune type in the IMvigor210 cohort.

D-E. Differences in TMB (D) and neoantigen burden (E) between WM_Score-high (red) and -low (blue) groups in the IMvigor210 cohort. Wilcoxon test was used to assess the difference. The boxes indicate the median ± 1 quartile, with the whiskers extending from the hinge to the smallest or largest value within 1.5× IQR from the box boundaries
